# An Eye-Movement Study of relational Memory in Adults with Autism Spectrum Disorder

**DOI:** 10.1007/s10803-017-3212-3

**Published:** 2017-07-07

**Authors:** Melanie Ring, Dermot M. Bowler, Sebastian B. Gaigg

**Affiliations:** Autism Research Group, Department of Psychology, City, University of London, Rhind Building, Northampton Square, London, EC1V 0HB UK

**Keywords:** Implicit and explicit memory, Relational memory, Autism Spectrum Disorder, Eye movements, Encoding and retrieval

## Abstract

Persons with Autism Spectrum Disorder (ASD) demonstrate good memory for single items but difficulties remembering contextual information related to these items. Recently, we found compromised explicit but intact implicit retrieval of object-location information in ASD (Ring et al. Autism Res 8(5):609–619, 2015). Eye-movement data collected from a sub-sample of the participants are the focus of the current paper. At encoding, trial-by-trial viewing durations predicted subsequent retrieval success only in typically developing (TD) participants. During retrieval, TD compared to ASD participants looked significantly longer at previously studied object-locations compared to alternative locations. These findings extend similar observations recently reported by Cooper et al. (Cognition 159:127–138, 2017a) and demonstrate that eye-movement data can shed important light on the source and nature of relational memory difficulties in ASD.

## Introduction

Besides the well-known difficulties in the areas of social interaction, communication, and flexibility in behaviour (American Psychiatric Association 2013), individuals with Autism Spectrum Disorder (ASD) show a characteristic cognitive profile with strengths and difficulties in areas, such as Theory of Mind (Baron-Cohen et al. 1985; Bowler et al. 2005; Frith and Frith 2003), perception (Frith and Happé 1994; Happé 1999; Mottron and Burack 2001; Mottron et al. 2006; Plaisted et al. 2006, 1998), attention (Allen and Courchesne 2001), executive functions (Hill 2004a, b), and memory (Boucher and Bowler 2008; Boucher et al. 2012). The present study focuses on the last of these domains, where evidence demonstrates a pattern of relatively preserved memory for single units of information (*item memory*), but difficulties in relating these units to one another, or to their spatial and temporal context (*relational memory*; see Bowler et al. 2011; Gaigg and Bowler 2012 for reviews). There are some inconsistencies in this literature, however, with some studies suggesting that item memory can be a source of difficulty for individuals with ASD (Cooper et al. 2015; Ring et al. 2016; Solomon et al. 2016), whilst others have shown preserved memory for relational context information (Gras-Vincendon et al. 2007; Lind et al. 2014a; Maister and Plaisted-Grant 2011; Souchay et al. 2013). A number of factors likely contribute to these discrepancies, including the extent to which tasks rely on executive function-related learning strategies (see Solomon et al. 2016), and whether to-be-remembered items are studied in isolation or in the context of other items (e.g., Ring et al. 2016). Another factor that may be involved is the extent to which test performance might be supported by implicit as well as explicit memory for the studied material. Memory difficulties in ASD tend to be more evident in tests of *explicit memory* that require the active retrieval of studied information (Tulving 2002), whereas *implicit memory*, which operates outside conscious awareness, tends to be preserved (Tulving 2002; Bowler et al. 1997; Gardiner et al. 2003; Renner et al. 2000). It is therefore possible that discrepancies in findings concerning *explicit* memory in ASD are partly due to differences in the role that *implicit* memory might play in different memory paradigms.

In a study (Ring et al. 2015) that required 25 ASD and 26 age and ability matched typically developing (TD) adults to remember object-locations in pictures of rooms, we have recently found that ASD adults only experienced difficulties in explicitly remembering object-location relations, whereas their implicit memory for the same material was preserved (i.e., they were as likely as TD participants to place objects into studied locations, when explicitly asked to choose new locations). This finding is important because it suggests that relational memory difficulties in ASD arise primarily because of difficulties in retrieving rather than encoding relational information, which has important implications for how memory difficulties might be alleviated in ASD (i.e., by providing support at retrieval; see Bowler et al. 2004). Unfortunately, however, the conclusions drawn from that study were somewhat tempered by ceiling performance in some individuals and floor performance in others. Moreover, the conclusions were at odds with other studies (e.g., Gaigg et al. 2008, 2015), which had suggested that atypical encoding processes do contribute to memory difficulties for relational information in ASD. For instance, Gaigg et al. (2008) asked participants to study lists of words either with no specific encoding instructions, or with instructions that emphasised item-specific (rate each word on pleasantness) or relational (sort words into categories) information. During subsequent free recall of these lists, the ASD and comparison groups performed similarly following the item-specific encoding instructions but not following the relational or no encoding instructions, where the ASD group performed significantly worse. Since the retrieval conditions were identical across conditions, the authors concluded that the encoding of relational information is a source of difficulty for individuals with ASD.

During our recent object-location memory study referred to above, we had the opportunity to collect eye-movement data for a sub-sample of the participants. These data will be the focus of the current paper because eye-movement data can shed unique light on the encoding and retrieval processes involved in implicit and explicit memory for relational information (e.g., Hannula and Ranganath 2009; Ryan et al. 2000; Ryan and Villate 2009). For example, experiments comparing participants’ eye-movements when looking at scenes that have been manipulated or left unchanged with respect to an earlier study phase demonstrate reliable relational memory effects whereby participants fixate manipulated areas of the scenes more (e.g., Ryan et al. 2000). These *eye-movement-based memory effects* operate below the level of awareness (Ryan et al. 2000), and are evident long before participants give an explicit response (Hannula et al. 2007), or even when no explicit response is required (Hannula et al. 2007). Thus, gaze behaviour during retrieval can give insight into implicit memory for relational information. In addition, monitoring gaze behaviour during encoding can reveal how the allocation of attention during study contributes to subsequent relational memory. For example, previous research has shown that the frequency of fixations at encoding is related to overall memory accuracy at test (Molitor et al. 2014; Olsen et al. 2016; Pertzov et al. 2009), and that memory for contextual information is specifically related to a pattern of fixations that is characterised by tightly clustered rather than more evenly spread fixations at encoding (Sharot et al. 2008). Another study has shown that more frequent and longer fixations to objects that are laid out on various surfaces in a three-dimensional space is associated with increased memory for the objects and their specific locations, but not with a more general knowledge about the spatial layout that was presented (Shih et al. 2012). The authors argued that this pattern of results suggests that individuals form a cognitive map of the general layout of their environment within a few fixations and that further fixations aid memory for the specific object-location relations rather than this general layout (Hollingworth and Henderson 2002; Shih et al. 2012). The mechanism of forming cognitive maps has been suspected to function abnormally in autistic individuals (Lind et al. 2013, 2014a; Ring et al. 2017), and to contribute to their memory difficulties (Bigham et al. 2010; Bowler et al. 2004, 2014; Cooper et al. 2015; Gaigg et al. 2014; Lind et al. 2014a, b; Ni Chuileann and Quigley 2013; Poirier et al. 2011; Ring et al. 2015, 2016; Terrett et al. 2013; Wojcik et al. 2013) and problems with spatial navigation (Lind et al. 2013, 2014a; Pellicano et al. 2011).

Only a few studies to date have examined memory processes in ASD through eye-movement data, with the majority examining memory for faces that is not the focus of the current paper (see Snow et al. 2011; Chawarska and Shic 2009; Hedley et al. 2012). Two studies, however, have focussed on the relation between encoding eye-movements and later memory for non-face stimuli in ASD. Specifically, Loth et al. (2011) asked participants to read stories and then look at scenes with objects that were either relevant, irrelevant, or neutral in relation to the stories. ASD participants recalled fewer story-relevant objects than TD participants, and eye-movement data pointed to reduced attention to story-relevant information during the initial period of scene viewing. In contrast, Cooper et al. (2017a) did not find a between-group difference in the number or spatial distribution of fixations at encoding when presenting participants with images of scenes under incidental and intentional learning conditions. When participants were asked to discriminate previously studied from similar lure scenes at test, however, ASD participants’ memory for the scenes was significantly reduced independent of the encoding instruction. Closer examination of the association between gaze data and behavioural performance furthermore showed that while retrieval success was related to the number and spatial distribution of encoding fixations in TD individuals, no such relation was found for persons with ASD. In addition, the extent to which the distribution of fixations at encoding matched the distribution at retrieval was associated with scene memory in TD participants but again not in ASD participants, leading the authors to conclude that scene memory difficulties in ASD arise primarily at the stage of retrieval. These studies show how valuable the measurement of eye-movements can be in the context of memory research in ASD to disentangle the contribution of encoding and retrieval processes.

Given the observations by Loth et al. (2011) and Cooper et al. (2017a), the first aim of this study was to examine whether differences in the allocation of attention at encoding may contribute to object-location memory difficulties in ASD. The second aim was to test the prediction that gaze patterns at retrieval would reveal evidence of relational memory difficulties in ASD, with reduced viewing of previously studied compared to non-studied object locations vis-a-vis the comparison group. If this prediction is confirmed, it would indicate that eye-movements could provide a viable method for investigating memory processes in younger and less able individuals with ASD in future studies.

## Method

### Participants

Of the 51 participants originally involved in our recent object-location memory study (Ring et al. 2015), eye-movement data were gathered for 23 TD (17 men) and 25 ASD (20 men) individuals. The data for five (all men) TD and five (all men) ASD individuals were excluded from the analyses because more than 30% of trials either during encoding, retrieval or both included less than 25% of valid raw data samples.^1^ This criterion was derived from an inspection of the data where it clearly separated participants for whom data quality was overall poor (*M* = 37% of invalid raw data samples) from those for whom it was excellent (*M* = 6% of invalid raw data samples). The final sample of 18 TD (15 men) and 20 ASD individuals (16 men) were matched on chronological age (CA), Verbal (VIQ), Performance (PIQ), and Full-scale Intelligence Quotient (FIQ), as measured by the third edition of the Wechsler Adult Intelligence Scale (WAIS-III^UK^; The Psychological Corporation 2000; see Table 1). All ASD participants had received their diagnosis according to DSM-IV-TR criteria (American Psychiatric Association 2000) from qualified clinicians in the UK National Health Service, and assessments with the Autism Diagnostic Observation Schedule (ADOS; Lord et al. 1989) and the Autism-Spectrum Questionnaire (AQ; Baron-Cohen et al. 2001) further corroborated difficulties in the areas of social-communication and repetitive behaviours that constitute the hallmark of the diagnosis.^2^ TD participants reported no personal or family history of psychiatric disorders or use of psychotropic medication. All participants were native English speakers. Informed consent was obtained from all individuals prior to the study and they were reimbursed for their time and travel expenses with standard university fees. The study was approved by City, University of London’s ethics committee and the procedures outlined below adhere to the ethical guidelines set out by the British Psychological Society.

**Table 1:** Participant characteristics for persons with autism spectrum disorder (ASD) and typically developing (TD) individuals

	ASD (16m, 4f)		TD (14m, 4f)				
Measure	*M*	*SD*	*M*	*SD*	*t* (37)	*p*	Cohen’s *d*
Age (years)	40.96	13.08	39.96	12.67	0.24	.81	0.08
VIQ^a^	107	16.06	113	11.70	1.49	.15	0.43
PIQ^b^	107	16.45	109	10.57	0.39	.70	0.14
FIQ^c^	107	16.63	112	11.39	1.05	.29	0.35
AQ^d^	32.68	5.38	15.28	15.28	5.52^e^	.00	3.19
ADOS-C^f^	2.11 (0–4)	1.18					
ADOS-RSI^g^	5.61 (3–10)	2.00					
ADOS-Total^h^	7.72 (3–12)	2.02					
ADOS-Im^i^	1.24 (0–2)	0.66					
ADOS-SB^j^	1.00 (0–3)	1.03					

For ADOS scores, range of scores is presented in brackets

^a^Verbal Intelligence Quotient (WAIS-III^UK^)

^b^Performance Intelligence Quotient (WAIS-III^UK^)

^c^Full-scale Intelligence Quotient (WAIS-III^UK^)

^d^AQ—Autism-Spectrum Quotient

^e^Here t (36)—all participants but one ASD individual had filled in the AQ

^f^ADOS—Communication subscale

^g^ADOS—Reciprocal Social Interaction subscale

^h^ADOS total score—Communication + Reciprocal Social Interaction

^i^ADOS—Imagination/Creativity subscale

^j^ADOS—Stereotyped Behaviours and Restricted Interests subscale

### Materials and Procedure

**Fig. 1:**
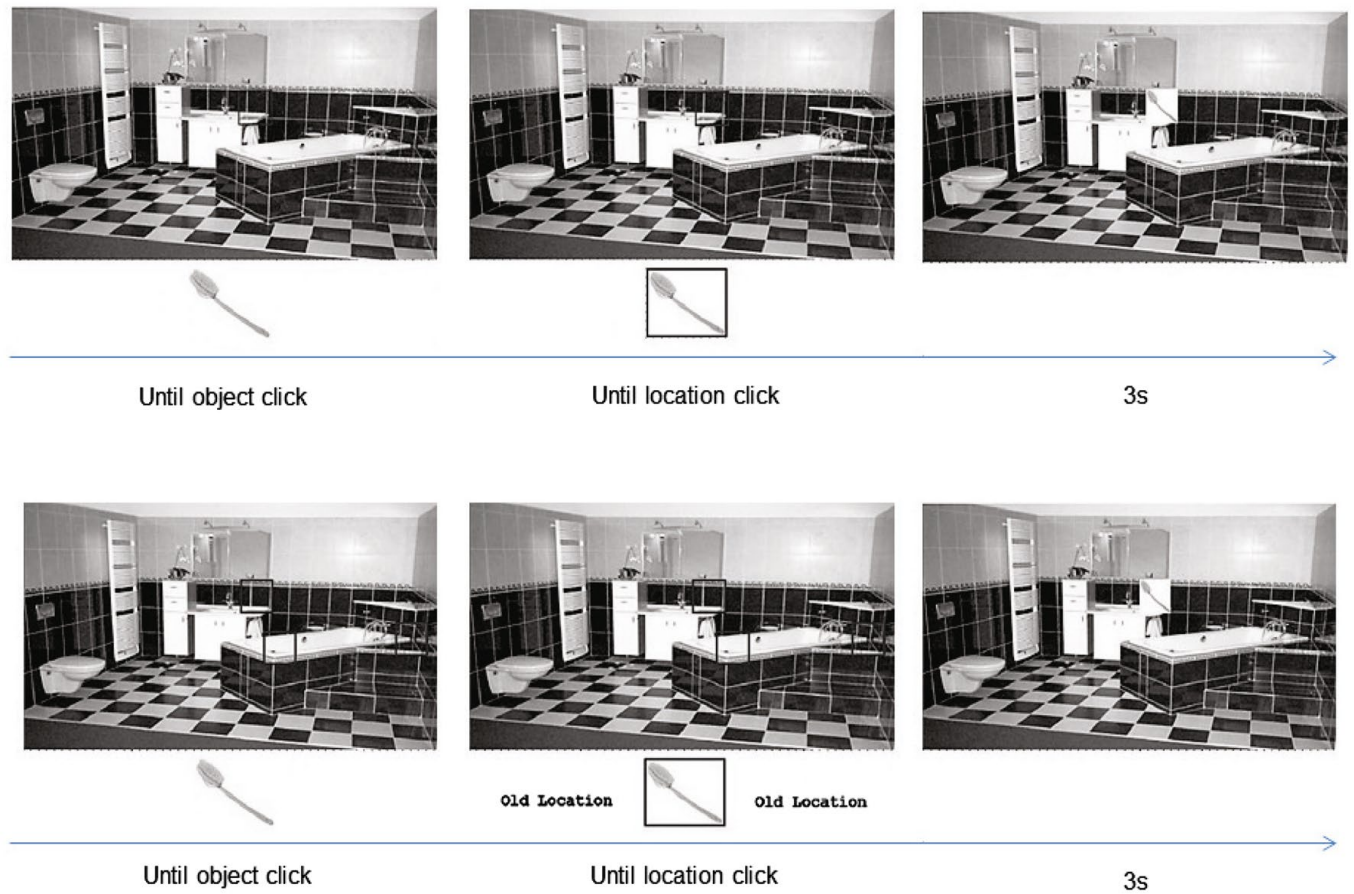
Examples of study phase (*top*) and test phase (*bottom*). Previously published in Ring et al. (2015) and with permission taken

The experimental materials and procedure are described in detail in Ring et al. (2015). Briefly, participants were asked to remember the locations of a series of 24 objects that were presented in unique locations of six rooms (i.e., four objects per room). Pictures of these rooms were presented to cover 80% of a standard desktop monitor, and on each trial a context appropriate object (e.g., a bar of soap for a bathroom) appeared below the room picture, whilst a to-be-remembered target location for this object was highlighted in the room picture with a red frame. Participants needed to click on the object, and then on the highlighted location, which resulted in the object appearing in that location for 3 s before the next object was presented underneath the room picture (see Fig. 1 top).

Immediately following the 24 study trials, participants were shown the room pictures again. On each test trial, an object appeared underneath the room that the participant had either studied or not, and three room locations were highlighted. Participants were either asked to put the object back into the location where it was studied (*include trials*), to choose one of the new locations (*exclude trials*) or to simply pick one of the locations if they could not remember having seen the object. Similar to the study trials, participants responded by first clicking on the object, and then on a location, which was followed by the display of the object in that location for 3 s before the next trial (see Fig. 1 bottom). The inclusion of both studied and novel objects during this test phase allowed for the estimation of response biases for different locations. Throughout the task, participants were asked to name the objects, and to describe their locations to control for verbal mediation strategies (Williams et al. 2012).

### Eye-Movement Recording and Analyses

Eye-movements were monitored throughout encoding and retrieval using a Tobii TX300 with a sampling rate of 120 Hz. Participants sat about 65 cm away from the 23″ screen. The eye-tracker did not require any head mounted equipment or a chin rest as it can accommodate head movements within a 37 cm × 17 cm plane at the 65 cm viewing distance. We nevertheless asked participants to sit still in a comfortable position throughout the study. The data were gathered following a standard five-point calibration procedure and the experiment was presented using the Tobii extension for E-prime, which derives information about the location of fixations using positional information from both eyes. Customised Matlab routines extracted the durations, latencies, and co-ordinates of all fixations lasting a minimum of 100 ms from the raw data (the spatial distribution constraint for fixations was approximately 0.7° of visual angle). For the *encoding* phase, fixation data were analysed throughout the duration of each trial, including the period during which the object was presented below the scene picture and the period during which it appeared in its target location. Regions of interest (ROIs) were defined for the room picture as a whole as well as for the object and the target location, which both measured 2.4 cm × 2.1 cm (2.1° × 1.8° of visual angle) for all trials. The duration of time participants spent looking at the scene, the object, and the target location was computed for each trial and then averaged across the trials that participants subsequently gave correct vs. incorrect retrieval responses for. For the *retrieval* phase, the average duration of time participants spent looking at the target (previously studied) and the new locations were computed and averaged across include and exclude trials. The analysis here focussed on the part of each trial that lasted from the appearance of the instructions until the selection of the location by the participant, since this part corresponds to the period of active retrieval. Our analyses focused on overall viewing durations because this measure was thought to reflect most closely how much time participants spent encoding and/or retrieving relevant information and because it would allow us to examine potential group differences in overall encoding/retrieval times.

## Results

Results were analysed using Chi-Squared tests for nominal data, independent samples t-tests, repeated measures ANOVAs and bivariate correlations. In the ANOVA analyses, the Greenhouse Geisser correction (GGC) was applied in cases where the Sphericity assumption was violated. The significance level was chosen at 0.05 for all tests and post hoc tests were calculated for significant differences. Cohen’s *d* and partial Eta-Squared are reported as effect size measures.

### Overt Response Accuracy

The behavioural results of this sub-sample of participants from our original study demonstrated the same pattern of results as previously reported in Ring et al. (2015). Briefly, ASD participants showed particular difficulties replacing an object into its old location (*p* < .01, Cohen’s *d* = 0.93), but they performed similarly to TD individuals, when choosing a new location (*p* = .37; Cohen’s *d* = 0.30), *F*(1,36) = 7.46, *p* < .05, η_p_^2^ = 0.17. This resulted in attenuated explicit, *t*(27.32) = 2.83, *p* < .01, Cohen’s *d* = 0.90, but preserved implicit, *t*(27) = 0.21, *p* = .83, Cohen’s *d* = 0.08, relational memory indices.^3^ It is also important to note that response times did not differ significantly between groups for either the encoding, *t*(36) = 0.95, *p* = .35, Cohen’s *d* = 0.31, or the retrieval phase, *F*_max_ < 2.38, *p*_min_ > .10, η_p_^2^_max_ < 0.07, making it unlikely that the results reported below are simply a reflection of group differences in processing speed.

### Eye-Movement Data: Encoding

**Fig. 2:**
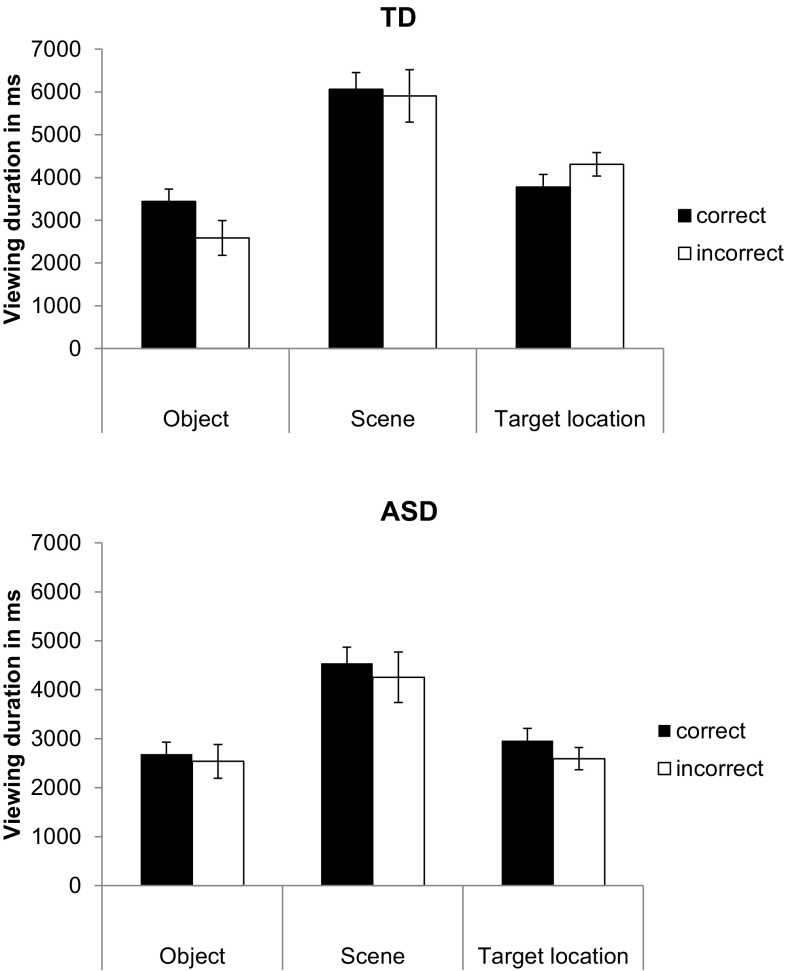
Viewing duration in ms for ASD and TD groups during encoding, as a function of region of interest: Object, Scene, and Target location separating data for trials that participants responded to correctly vs. incorrectly during the subsequent test. *Panel A* (*top*) illustrates the data for TD and *Panel B* (*bottom*) illustrates the data for ASD individuals. The data are presented as mean ± SEM

Data quality during encoding was excellent and there were no group differences in terms of the percentage of invalid raw data samples (ASD: *M* = 8.0%, *SD* = 5.1; TD: *M* = 8.1%, *SD* = 4.7; *t* = 0.62; *df* = 36; *p* = .96). To establish whether there were group differences in the allocation of attention during encoding as a function of subsequent retrieval success, a 2 (Group [ASD, TD]) × 2 (Subsequent memory [Correct, Incorrect]) × 3 (ROI [Object, Scene, Location]) repeated measures ANOVA was carried out. This demonstrated a significant main effect of ROI, *F*(2,54) = 53.22, *p* < .0001, η_p_^2^ = 0.66, whereby participants viewed the scene significantly longer than the target location (*p* < .0001, Cohen’s *d* = 1.36) or the object (*p* < .0001, Cohen’s *d* = 1.70). The data further revealed a Group × ROI interaction, *F*(2,54) = 3.28, *p* < .05, η_p_^2^ = 0.11, which was further qualified by a trend-level Group × Subsequent memory × ROI interaction, *F*(2,54) = 2.80, *p* = .07, η_p_^2^ = 0.09. Follow-up comparisons showed that this interaction was the result of a lack of difference in viewing duration of the object in the ASD group between subsequently correct vs. incorrect recognition responses (*t* = 0.52; *df* = 16; *p* = .61, Cohen’s *d* = 0.11), whereas TD individuals looked significantly longer at the objects (*t* = 3.12; *df* = 11; *p* < .05, Cohen’s *d* = 0.75) for which they subsequently remembered the locations. This pattern is illustrated in Fig. 2 and is in line with the recent observations of Cooper et al. (2017a). It is important to note that for this analysis of subsequently correct vs. incorrect trials, only 12 TD (ten men, two women, *M*_age_ = 42.18 years, age range: 26–61) and 17 ASD (13 men, 4 women, *M*_age_ = 42.03 years, age range: 25–69) participants could be included since the remaining participants made no errors during retrieval. The smaller groups in this analysis, however continued to be matched in terms of gender, *X* ^2^ = 0.31, *p* = .58, CA, VIQ, PIQ, and FIQ, *t*_max_ < 1.08, *p*_min_ > .33, Cohen’s *d*_max_ < 0.39.

### Eye-Movement data: Retrieval

**Fig. 3:**
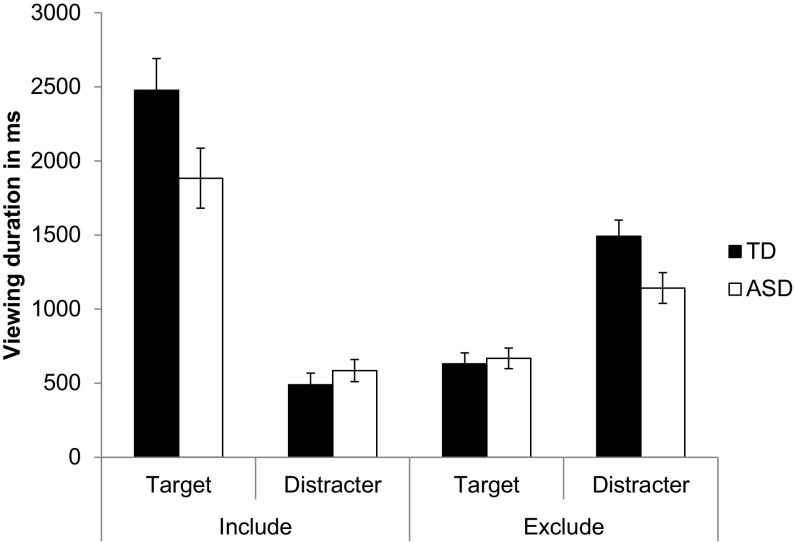
Viewing duration in ms for ASD and TD groups during retrieval as a function of retrieval instructions: Old location (include condition) versus New location (exclude condition), and region of interest: Target (studied) versus Distracter (unstudied/new) location. The data are presented as mean ± SEM

Data quality during retrieval was excellent and there were no group differences in terms of the percentage of invalid raw data samples (ASD: *M* = 10.0%, *SD* = 5.6; TD: *M* = 8.4%, *SD* = 5.0; *t* = 1.34; *df* = 36; *p* = .19). A 2 (Group [ASD, TD]) × 2 (Instruction [Include, Exclude]) × 2 (ROI [Target, Distracter]) repeated measures ANOVA of the retrieval data, set out in Fig. 3, showed significantly longer viewing times during include than exclude conditions, *F*(1,36) = 41.52, *p* < .0001, η_p_^2^ = 0.54, and on the target compared to the distracter locations, *F*(1,36) = 65.52, *p* < .0001, η_p_^2^ = 0.65. The data were further qualified by a significant Instruction x ROI interaction, *F*(1,36) = 151.00, *p* < .0001, η_p_^2^ = 0.81, and a three-way Group × Instruction × ROI interaction, *F*(1,36) = 8.19, *p* < .01, η_p_^2^ = 0.19. Follow-up comparisons showed that participants looked overall longer at target compared to the distracter locations during include trials (*p* < .0001, Cohen’s *d* = 2.33), and longer at distracter than target locations for exclude trials (*p* < .0001, Cohen’s *d* = 1.64), with this difference being less pronounced in the ASD group, who looked at the target location less than the TD group during the include trials (*t* = 2.01, *df* = 36, *p* = .052; Cohen’s *d* = 0.65) and less at the distracter locations during exclude trials (*t* = 2.33, *df* = 36, *p* < .05, Cohen’s *d* = 0.75; see Fig. 3).

### Eye-Movement Data: Unstudied Objects

As noted earlier, some objects presented at test had not been studied and, therefore, served to estimate possible response biases for choosing certain locations. The analysis of eye-movement data for these trials indicated no differences between groups that could account for the above reported three-way interaction. Specifically, a 2 (Group [ASD, TD]) × 2 (Instruction [Include, Exclude]) × 2 (ROI [Target, Distracter]) repeated measures ANOVA showed no significant main effects or interactions, *F*_max_ < 1.19, *p*_min_ > .28, η_p_^2^_max_ < 0.04.

### Association Between Behavioural and Eye-Tracking Measures at Retrieval

**Fig. 4:**
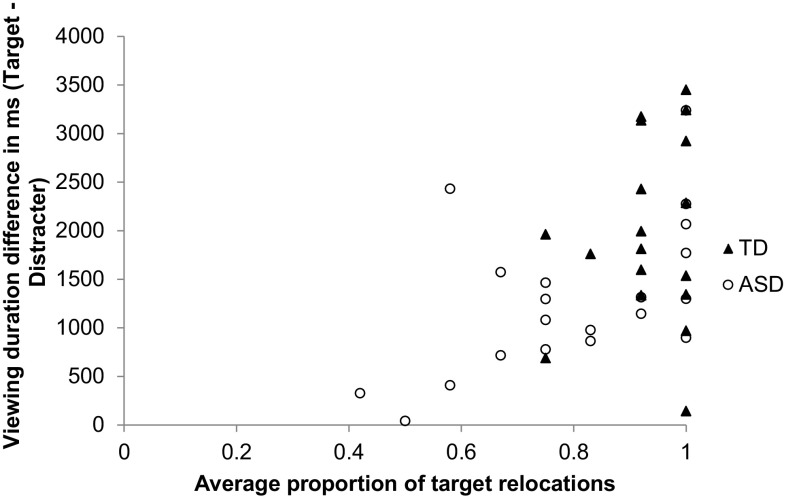
Association between the proportion of target location selections for the include condition and the difference in fixation durations between the target and distracter locations for the include condition. The correlation illustrates that a greater propensity to look at the target versus the distracter locations is predictive of the retrieval of the target location in the overt behavioural response

Figure 4 plots the difference in fixation durations between target and distracter locations for the include condition (i.e., the condition where participants were asked to choose the previously studied target location) against the proportion of times participants selected the target location as their answer for the include condition (i.e., the proportion of times they gave a correct answer for this condition). The strong correlation between these variables (*r* = 0.48, *p* < .005) confirms that eye-tracking data during retrieval can provide valuable insight into memory processes.^4^ Overall, these data show that participants’ tendency to choose previously studied object locations can be predicted on the basis of how much they attend to such locations and by how much they avert attention from distracter locations.

## Discussion

Following our recent demonstration of impaired explicit but preserved implicit memory for object–location relations in ASD (Ring et al. 2015), the present study reports on eye-tracking data that we had the opportunity to gather for a sub-sample of the participants involved in that study. Since it is often difficult to disentangle encoding from retrieval processes in behavioural measures of memory, and because it remains unclear to what extent atypical encoding might contribute to atypical retrieval of relational information in ASD, the first aim of this study was to examine how individuals with and without ASD allocate their attention when trying to remember object–locations in complex scenes. The results demonstrated that, ASD and TD participants did not differ overall in how much time they spent looking at the scenes and object locations they were asked to remember. However, when taking subsequent retrieval success into account, it became apparent that encoding-related viewing times differentiated between subsequently remembered versus forgotten object locations only in the TD but not the ASD group. Specifically, TD participants spent more time looking at objects for which they subsequently remembered the scene locations compared to objects for which they forgot the scene locations. This pattern, which replicates the findings of Shih et al. (2012), was not apparent in the ASD group. Cooper et al. (2017a) recently reported very similar findings and argued that the absence of a subsequent memory effect in ASD combined with a lack of group differences in overall looking behaviour during encoding suggests that memory retrieval rather than encoding processes are compromised in ASD. This conclusion, however, assumes that ASD participants encode the information they look at in the same way as TD participants, which is at odds with the finding that memory difficulties in ASD vary as a function of encoding condition even when retrieval conditions are held constant (Gaigg et al. 2008, 2015; Toichi and Kamio 2002). The absence of a subsequent memory effect in encoding eye-movements in ASD may therefore also be a reflection of atypical encoding processes such as difficulties in relational binding (e.g., Bowler et al. 2011, 2014) or the use of executive function-related encoding strategies (e.g., Solomon et al. 2016; Southwick et al. 2011). Future studies could shed further light on these issues by systematically examining the effects of various encoding manipulations on eye-movement related subsequent memory effects (see Cooper et al. 2017a).

A second aim of the current study was to establish whether the overt behavioural difficulties individuals with ASD experience in explicitly retrieving relational information might also be revealed in the gaze behaviours of participants. The results suggest that this is indeed the case. Specifically, persons with ASD looked significantly less at the target locations during include trials and less at distracter locations during exclude trials, indicating that reduced memory for object–location relations in ASD can be revealed not only through behavioural tests that require an overt response but also through eye-movement-based data that can be passively recorded (Althoff and Cohen 1999; Ryan et al. 2007). This finding extends evidence of impaired relational memory in ASD to a measure that operates largely outside of conscious awareness and that could therefore be suitable for examining memory functions in under-researched ASD populations, such as individuals with lower intellectual and/or language abilities, or very young individuals with ASD. Expanding research efforts in relation to memory processes to these groups will be critical for the formulation of developmental accounts that try to specify what role atypical memory function might play in the aetiology of ASD in general, and in relation to the heterogeneity of the disorder in particular. For instance, Boucher et al. (2008) have argued that the patterning of memory strengths and difficulties in different individuals on the autism spectrum might hold the key for understanding the heterogeneity in language development across the spectrum, and eye-tracking methods may prove useful in this context.

The data presented here allow for some speculations about brain regions underlying the memory difficulties observed in ASD. The expression of memory in eye-movements has been found to be related to activity in the hippocampus and prefrontal cortex (PFC; Hannula and Ranganath 2009), and attentional processes underlying differences found in eye-movements during encoding, were found to be related to functions of the medial temporal lobe, PFC, as well as the parietal cortex (Cabeza et al. 2008). Therefore, the current data further support the suggested role of hippocampal–frontal processes in the memory difficulties in ASD (Bowler 2007; Bowler et al. 2011; Minshew and Goldstein 1998). A recent fMRI study by Cooper et al. (2017b) shows direct evidence for this proposal in presenting reduced connectivity in hippocampal–frontal networks during episodic memory retrieval.

We must acknowledge the modest sample size as a limitation of the present study, and recognise that some caution is warranted in the interpretation of the results, given that the number of analyses across this and the associated Ring et al. (2015) paper raise concerns over the possibility of Type 1 errors. Another limitation of the current study is the small number of 24 trials for each participant. Splitting up the data by conditions and type of response leaves few trials for the analyses making the results prone to bias. Notwithstanding that further replication will be important, the observations contribute to the literature by extending the recent observations of Cooper et al. (2017a) in demonstrating that eye-movement data can provide unique insights into the memory difficulties associated with ASD. Moreover, the observations develop the empirical foundations for future studies to examine memory processes in more representative samples, including younger individuals and individuals with language and/or intellectual impairments that remain shamefully underrepresented in the literature.

## Footnotes

1 The Tobii provides a quality index for each data sample for each eye, which ranges from 0 (ideal) to 4 (eye cannot be detected). We considered raw samples to be invalid if there was a signal loss (4) in either eye.

2 Because of time constraints on the day of testing, we were not able to carry out the ADOS with three individuals, and the observations for one participant resulted in below-threshold algorithm scores. Since clinical records provided clear evidence of their diagnosis, however, all participants were retained in the analyses.

3 As explained in more detail in Ring et al. (2015), explicit and implicit indices were calculated on the basis of Jacobys’ process dissociation procedure (Jacoby 1991, 1998), which assumes imperfect performance in both the include and exclude conditions. Because some participants made no errors in one of the conditions, the indices could only be calculated for 12 TD and 17 ASD participants in the current study.

4 Examining the correlations separately for the two groups showed no correlation for the TD (*r* = 0.18, * p* = .47) but a strong positive correlation for the ASD group (*r* = 0.55, *p* < .05). This apparent difference is difficult to interpret given the very restricted range of very good behavioural performance in the TD group (see Fig. 4) and will therefore not be discussed further.
